# Outcomes of Percutaneous Cholecystostomy as a Bridging or Definitive Treatment for Acute Cholecystitis

**DOI:** 10.7759/cureus.100668

**Published:** 2026-01-03

**Authors:** Umer Qureshi, Khurram Siddique

**Affiliations:** 1 General and Colorectal Surgery, The Royal Oldham Hospital, Oldham, GBR

**Keywords:** acute cholecystitis, catheter duration, cholecystectomy alternative, clinical outcomes, percutaneous cholecystostomy

## Abstract

Percutaneous cholecystostomy (PC) is a minimally invasive intervention for managing acute cholecystitis in patients unfit for immediate cholecystectomy. Although it is widely used, its outcomes and factors influencing clinical success are not well understood.

Objectives

The goal of this study was to assess the outcomes of PC by examining various factors such as gender, type of cholecystitis, and procedure timing to correlate with symptom improvement, mortality, and the need for additional medical interventions.

Materials and methods

A cross-sectional study was conducted at The Royal Oldham Hospital UK, from December 2019 to June 2024. A total of 70 patients who underwent PC for acute cholecystitis were included. Data on patient demographics, type of cholecystitis, timing of the procedure, duration of catheter placement, and clinical outcomes were collected and analysed using appropriate statistical tests.

Results

Seventy patients (mean age 53.70±16.48 years; catheter duration 17.70±6.73 days) were analysed. Symptom resolution occurred in 61 (87.1%) patients and failed in nine (12.9%). Among middle-aged adults, 19 (90.4%) improved versus 42 (85.7%) older adults (p=0.766). In men, 36 (90.0%) cases were resolved, whereas 25 resolved in women (83.3%) (p=0.410). Bile leakage occurred in five (7.1%) patients, of which two (9.1%) were acalculous and three (6.3%) calculous (p=0.668). Mortality occurred in two patients (10.2%) in delayed PC and none (0.0%) in early PC (p=0.05). Additional interventions occurred in 32 (45.7%) patients: four (33.3%) short-term, nine (36%) moderate term and 19 (57.5%) long-term (p=0.945). PC led to high success (87.1%), low complications (7.1%), and low mortality (10.2%).

Conclusion

PC is an effective and safe way of managing acute cholecystitis in high-risk patients, with high symptom resolution and low complication rates. Our study findings did not reveal any significant effect of procedural and patient factors on outcomes. Appropriate patients and individually tailored management strategy is the key to advise best possible outcomes and long-term management.

## Introduction

Percutaneous cholecystostomy (PC) has been developed for the management of acute cholecystitis in high-risk patient groups, particularly elderly patients and those with serious concomitant diseases [1]. This minimally invasive procedure involves the use of a percutaneous catheter to decompress the inflamed gallbladder, which is a safer alternative to emergency cholecystectomy in patients deemed unfit for surgery. Nevertheless, although short-term outcomes are well documented, evidence regarding long-term outcomes, such as recurrence, morbidity, and mortality [2], is lacking.

Poor outcomes are predicted by factors such as calculous cholecystitis, longer catheter dwell time, and severe systemic inflammation [3,4]. Furthermore, patients who are deemed unfit for surgical management and managed with PC alone have died at higher rates due to frailty and other comorbidities, such as cardiovascular disease and malignancy [5,6]. The insights gained from these data are essential in devising the treatment plan and counselling patients on their prognosis.

This study emphasises evidence by measuring the outcomes of PC as a standalone treatment. Focusing on acupuncture therapy, some evidence supports its efficacy in managing acute cholecystitis in critically ill patients; however, interval cholecystectomy is supported by other studies as a means of decreasing long-term complications and recurrence rates. Whether PC can be definitive or bridging therapy depends on the overall health and comorbidities of the patient, as well as life expectancy.

Although PC has improved with intervention, its outcomes, as well as the effects of patient and procedural factors, remain unclear. These variables must be understood for the best decision regarding treatment to prevent complications and improve patient outcomes. The gaps were addressed in this study through the evaluation of PC outcomes and factor analysis surrounding clinical success.

## Materials and methods

This cross-sectional study was conducted at The Royal Oldham Hospital. Retrospective data was collected from December 2019 to June 2024. This study evaluated PC as a bridging or alternative treatment of cholecystectomy in patients with severe cholecystitis.

The sample size was calculated using the prevalence of complications reported by Alvino et al. [6], where the complication rate was 7%, with a 95% confidence level and a margin of error (D) of 6%. This resulted in a required sample size of 70 patients. A non-probability consecutive sampling technique was used to recruit eligible patients who met the inclusion criteria.

Patients included in the study were those who underwent PC for the management of severe acute cholecystitis, as diagnosed based on clinical, radiological, and laboratory findings. Patients with concurrent malignancies or those who refused to go for PC were excluded from the study.

Data collection involved recording demographic information, such as age, gender, and comorbidities (e.g., diabetes, hypertension). Clinical variables, including the type of cholecystitis (calculous or acalculous), timing of the procedure (from admission to procedure), and duration of catheter placement (short-term or long-term), were also documented. Follow-up was conducted at regular intervals to assess symptom resolution, the need for additional interventions, the occurrence of complications (e.g., infection at the catheter site or bile leakage), and mortality during the study period.

All procedures were performed by experienced radiologists under ultrasound or CT guidance using standard sterile techniques. Patients were monitored by post-procedure for immediate complications and received appropriate management according to the hospital protocol.

Data was analysed using SPSS for Windows version 24 (IBM Corp., Armonk, NY). Continuous variables, such as age and duration of catheter placement, presented as means and standard deviations. Categorical variables, such as gender, type of cholecystitis, complications, need for additional interventions, and mortality, were presented as frequencies and percentages. The chi-square test was used to assess the association between categorical variables. A p-value of less than 0.05 was considered statistically significant.

## Results

The study included 70 patients, with a mean age of 53.70±16.48 years and a mean duration of catheter placement of 17.70±6.73 days.

Association between age and symptom resolution

Symptom resolution following PC was observed in 61 (87.1%) patients, while nine (12.9%) did not achieve resolution. When analysed by age group, among middle-aged adults (42-52 years), 90.4% (19) achieved resolution, while 10% (two) did not. In the older adult group (53 and older), 85.7% (42) showed symptom improvement, whereas 14.2% (seven) continued to experience symptoms. The Pearson chi-square test revealed no significant association between age group and symptom resolution, suggesting that age does not play a significant role in clinical improvement after PC (χ²(1, N=70)=0.089, p=0.766, Cramér’s V=0.036 (small effect)). Although younger patients exhibited slightly higher resolution rates, the differences across age groups were minor and not statistically meaningful. These findings indicate that PC is equally effective for symptom resolution in different age groups (Table 1).

**Table 1:** Association Between Age and Symptom Resolution

Age Group	Symptom Resolution: Yes (%)	Symptom Resolution: No (%)	Total (N)	p-value
Adults (42-52 years)	19 (90.4%)	2 (9.6%)	21	0.766
Adults (53 and older)	42 (85.7%)	7 (14.3%)	49	
Total	61 (87.1%)	9 (12.9%)	70	

Gender and symptom resolution

The association between gender and symptom resolution demonstrated a higher resolution rate among men, with 36 (90.0%) achieving resolution and four (10.0%) continuing to have symptoms. Among women, 25 (83.3%) experienced resolution, while five (16.7%) did not. The Pearson chi-square test showed no significant association between gender and symptom resolution (χ²(1, N=70)=0.680, p=0.410, Cramér’s V = 0.099 (small effect)), indicating that gender did not significantly influence symptom outcomes following PC (Table 2).

**Table 2:** Association Between Gender and Symptom Resolution

Gender	Symptom Resolution: Yes (%)	Symptom Resolution: No (%)	Total (N)	p-value
Male	36 (90.0%)	4 (10.0%)	40	0.410
Female	25 (83.3%)	5 (16.7%)	30	
Total	61 (87.1%)	9 (12.9%)	70	

Type of cholecystitis and complications

Bile leakage was reported in five (7.1%) patients, while 65 (92.9%) remained complication-free. When analysed by type of cholecystitis, two (9.1%) patients with acalculous cholecystitis developed bile leakage compared to three (6.3%) with calculous cholecystitis. No significant association was found between type of cholecystitis and bile leakage (χ²(1, N=70)=0.183, p=0.668, Cramér’s V=0.051 (small effect)) (Table 3).

**Table 3:** Association Between Type of Cholecystitis and Complication (Bile Leakage)

Type of Cholecystitis	Bile Leakage: Yes (%)	Bile Leakage: No (%)	Total (N)	p-value
Acalculous Cholecystitis	2 (9.1%)	20 (90.9%)	22	0.668
Calculous Cholecystitis	3 (6.3%)	45 (93.8%)	48	
Total	5 (7.1%)	65 (92.9%)	70	

Timing of percutaneous cholecystostomy and mortality

The overall mortality rate following the timing of PC since admission was 10.2% (two), and 89.4%(17) patients survived in the delayed plan of PC after admission. Subsequently, mortality occurred in patients due to other medical causes, whereas no mortality (0.0%) was observed in the early PC procedure after admission, comparatively. Among those who underwent early PC, all 51 (100.0%) patients survived. These findings indicate that the timing of PC did not significantly impact patient survival, although mortality was observed exclusively in patients who had multiple comorbidities and died due to other medical conditions in the following years. The overall mortality rate remained low, and the difference was not statistically meaningful. These findings suggest that PC is a generally safe procedure with high survival rates (Pearson chi-square test: χ²(1, N=70)=3.76, p=0.05, Cramér’s V=0.232 (small-to-moderate effect)) (Table 4). 

**Table 4:** Association Between Timing of Procedure and Mortality

Timing of PC	Mortality: Yes (%)	Mortality: No (%)	Total (N)	p-value
Early Plan of PC	0 (0.0%)	51 (100.0%)	51	0.05
Delayed PC	2 (10.2%)	17 (89.8%)	19	
Total	2	68	70	

Association between duration of catheter placement and additional interventions

Among the 70 patients, 32 patients (45.7%) required further intervention, such as interval cholecystectomy following PC, while 38 (54.3%) did not. When assessed by duration of catheter placement, four patients (33.3%) in the short-term group (1-10 days), nine patients (36.0%) in the moderate-term group (11-20 days), and 19 patients (57.5%) in the long-term group (21-30 days) required additional procedures (interval cholecystectomy). Despite this, the majority of patients in each duration category did not undergo further intervention. Among them, eight (66.7%) patients in the short-term catheter placement group, 16 (64.0%) patients in the moderate-term group and 14 (42.4%) patients in the long-term group did not go for interval cholecystectomy. Statistical analysis demonstrated no significant association between catheter duration and the need for subsequent procedures (χ²(2, N=70)=0.112, p=0.945, with a small effect size (Cramér’s V=0.040)) (Table 5).

**Table 5:** Association Between Duration of Catheter Placement and Additional Interventions

Duration Group	Interval Cholecystectomy: Yes (%)	Interval Cholecystectomy: No (%)	Total (N)	p-value
Short-Term (1-10 days)	4 (33.3%)	8 (66.7%)	12	0.945
Moderate-Term (11-20 days)	9 (36.0%)	16 (64.0%)	25	
Long-Term (21-30 days)	19 (57.5%)	14 (42.4%)	33	
Total	32 (45.7%)	38 (54.3%)	70	

## Discussion

PC is a widely recognised alternative to cholecystectomy for the management of acute cholecystitis (AC) [1]. Ongoing research on the role of PC is focused on whether it is a definitive treatment or a bridge to interval cholecystectomy. For many patients, PC is temporary because it improves the patient’s condition until definitive surgical intervention can be safely performed. However, if surgery is not appropriate, PC alone may be adequate, as studies have demonstrated recurrence rates as low as 9%-23% with appropriate follow-up care [2,3,7-9].

The use of PC is beneficial, although potential risks exist. Approximately 10%-20% of cases report tube dislodgement, infection, or bile leaks, which may lead to extended hospital stays and increased morbidity. Additionally, recurrent cholecystitis and biliary complications may occur more frequently following PC alone compared with cholecystectomy, highlighting the importance of careful patient selection and long-term planning [4].

PC is associated with high technical success rates ranging from 99% to 100%, with rapid symptom relief in most patients [6]. In our study, symptom resolution following PC was observed in 87.1% of patients, consistent with prior findings [3,6]. Alvino et al. demonstrated a 91% resolution rate for acute calculous cholecystitis following PC placement [6]. Similarly, Bundy et al. reported a 100% technical success rate with clinical resolution in all patients [10]. These findings affirm PC as a reliable stabilising procedure for critically ill patients.

When analysed by gender, men demonstrated a slightly higher resolution rate (90.0%) than women (83.3%), although this difference was not statistically significant. This aligns with previous studies reporting no clear gender-based differences in outcomes following PC [3,11,12].

Although PC is generally safe, complications such as bile leakage remain a concern. In this study, bile leakage occurred in 7.1% of patients, with no statistically significant association between cholecystitis type and bile leakage. Alvino et al. reported a 7% complication rate, comparable to our findings [6]. 

The timing of PC relative to hospital admission was the most critical determinant of outcomes. Patients who underwent early PC experienced faster symptom resolution, shorter hospital stays, and improved clinical outcomes. This finding aligns with those of Grisotti et al. and Bhatt et al., who emphasised the benefits of early intervention [9,13-15].

Long-term outcomes depend on patient comorbidities and definitive management strategies. In our study, 20.0% of patients required additional interventions following PC, with no significant association between catheter duration and intervention rates. Interval cholecystectomy has been shown to reduce recurrent biliary events. Alvino et al. reported a reduction in recurrence from 21% to 7% following interval cholecystectomy [6]. Conversely, expectant management is associated with higher recurrence rates, reported at approximately 20% in long-term follow-up studies [7,11]. Ozyer reported an 87.2% long-term success rate for PC as definitive treatment in acute acalculous cholecystitis [16]. Other studies have similarly reported risks of infection, tube dysfunction, and biliary complications, underscoring the need for vigilant monitoring [10].

The timing of PC tube removal also influences recurrence and readmission rates. Di Martino et al. demonstrated significantly higher recurrence and readmission rates with early tube removal (<7 days) compared with delayed removal [17]. Park et al. recommended maintaining the tube for at least six weeks in high-risk patients to minimise recurrence [18].

Patient characteristics also influence outcomes. Although no significant association between age and symptom resolution was identified in this study, previous research has shown poorer outcomes in elderly and critically ill patients. Sanaiha et al. further demonstrated increased mortality and morbidity among patients with grade III cholecystitis treated with PC [8]. Huang et al. similarly identified comorbidity burden as a major predictor of prognosis following PC [19]. Lu et al. reported higher mortality among PC patients compared with cholecystectomy, with benefits primarily observed in high-risk populations [20].

This study has several limitations. Firstly, it was conducted at a single centre, which may restrict the generalisability of the results to other clinical settings with different patient populations or institutional protocols. Secondly, the sample size was modest, potentially limiting the statistical power to detect smaller effect sizes. Additionally, the retrospective nature of the study introduces inherent risks of selection bias and unmeasured confounding variables.

To confirm and extend these findings - particularly regarding the observed benefits of early decision-making for PC - well-designed, prospective, multicentre studies are warranted. Such investigations would enhance the external validity of our results and support the development of standardised clinical guidelines.

## Conclusions

PC was found to be an effective and safe intervention for acute cholecystitis, providing symptom relief in most patients regardless of age or gender. The procedure showed a low complication and mortality rate. The duration of catheter placement does not influence the need for additional interventions, suggesting that clinical decisions should be guided by patient-specific factors. Our study supports the use of PC as a safe and reliable alternative for high-risk surgical candidates to provide them the best symptom relief.
